# Spatial integration framework of solar, wind, and hydropower energy potential in Southeast Asia

**DOI:** 10.1038/s41598-022-25570-y

**Published:** 2023-01-07

**Authors:** Anjar Dimara Sakti, Pitri Rohayani, Nurusshobah Ainul Izzah, Nur Afrizal Toya, Pradita Octoviandiningrum Hadi, Thanti Octavianti, Wendi Harjupa, Rezzy Eko Caraka, Yunho Kim, Ram Avtar, Nattapong Puttanapong, Chao-Hung Lin, Ketut Wikantika

**Affiliations:** 1 Remote Sensing and Geographic Information Sciences Research Group, Faculty of Earth Sciences and Technology, Institut Teknologi Bandung, Bandung, 40132 Indonesia; 2 Center for Remote Sensing, Institut Teknologi Bandung, Bandung, 40132 Indonesia; 3 Ministry of Agrarian Affairs and Spatial Planning/National Land Agency, Jakarta, Indonesia; 4 Power Engineering Research Group, School of Electrical Engineering and Informatics, Institut Teknologi Bandung, Bandung, 40132 Indonesia; 5 Department of Geography and the Environment, University of the West of England, Bristol, UK; 6 National Research and Innovation Agency, Bandung, West Java 40135 Indonesia; 7 Department of Mathematical Sciences, College of Natural Science, Ulsan National Institute of Science and Technology, Ulsan, South Korea; 8 Faculty of Environmental Earth Science, Hokkaido University, Sapporo, 060-0810 Japan; 9 Faculty of Economics, Thammasat University, Bangkok, Thailand; 10 Department of Geomatics, National Cheng Kung University, Tainan, 70101 Taiwan

**Keywords:** Renewable energy, Climate-change mitigation, Environmental impact

## Abstract

Amid its massive increase in energy demand, Southeast Asia has pledged to increase its use of renewable energy by up to 23% by 2025. Geospatial technology approaches that integrate statistical data, spatial models, earth observation satellite data, and climate modeling can be used to conduct strategic analyses for understanding the potential and efficiency of renewable energy development. This study aims to create the first spatial model of its kind in Southeast Asia to develop multi-renewable energy from solar, wind, and hydropower, further broken down into residential and agricultural areas. The novelty of this study is the development of a new priority model for renewable energy development resulting from the integration of area suitability analysis and the estimation of the amount of potential energy. Areas with high potential power estimations for the combination of the three types of energy are mostly located in northern Southeast Asia. Areas close to the equator, have a lower potential than the northern countries, except for southern regions. Solar photovoltaic (PV) plant construction is the most area-intensive type of energy generation among the considered energy sources, requiring 143,901,600 ha (61.71%), followed by wind (39,618,300 ha; 16.98%); a combination of solar PV and wind (37,302,500 ha; 16%); hydro (7,665,200 ha; 3.28%); a combination of hydro and solar PV (3,792,500 ha; 1.62%); and a combination of hydro and wind (582,700 ha; 0.25%). This study is timely and important because it will inform policies and regional strategies for transitioning to renewable energy, with consideration of the different characteristics present in Southeast Asia.

## Introduction

Under Sustainable Development Goal 7, many countries have agreed to increase and distribute renewable energy sources, which made up only 11% of the total global energy supply in 2020^1,2^. With the global energy demand expected to increase by 50% between 2018 and 2050, strategies to increase the number of renewable energy sources to meet future energy demands have become more important than ever. Countries in Southeast Asia have been experiencing rapid economic and demographic growth over the last few decades, which has led to soaring energy needs. Unfortunately, fossil fuels account for more than half of the region's energy supply^3^. Southeast Asian countries have committed to target a 23% increase in renewable energy by 2025^4^. As areas that receive sunlight year-round and are dominated by numerous islands and mountains, countries in Southeast Asia have great potential for renewable energy. However, the main challenge in developing renewable energy is finding areas with high suitability for developing the infrastructure needed for sustainable power generation^5^. In addition, regulatory certainty, stable political and administrative coordination, thorough planning, and the identification of mature land constraints are needed to ensure that the energy tariffs in different regions are valued appropriately. Strategic renewable energy sources that have been developed in recent decades in the region include solar, wind, and hydropower. These sources have promising prospects for large-scale development to meet the region's renewable energy targets^4^ as well as to distribute energy to areas that do not yet have electricity^6^. Owing to the potential and constraints of developing sustainable energy infrastructure in Southeast Asia, a strategy is needed to identify the optimal locations for sustainable energy development in the region, for which this study seeks to contribute.

Remote sensing technology integrated with spatial analysis has been widely used to support decision making to determine the optimal locations for renewable energy infrastructure^7–9^. For example, to determine the optimal solar area, Lopez et al.^10^ used the MODIS remote sensing product to model solar radiation. Letu et al.^11^ estimated the values of surface solar radiation, clouds, and aerosols from Himawari-8 satellite measurements. Furthermore, Principe and Takeuchi^12^ conducted an assessment of potential solar photovoltaic (PV) energy in the Asia–Pacific region based on meteorological factors. After identifying the potential areas for solar energy using remote sensing, it is possible to select the areas with the highest optimal values for the construction of solar power infrastructure. In addition, spatial analyses have been conducted based on a multi-criteria approach related to solar PV placement^13–15^. For wind farms, Blankenhorn and Resch^16^ estimated the locations for potential wind energy in Germany based on the parameters of wind speed, land cover, slope, and protected area locations. Sah and Wijayatunga^17^ modeled the potential regions in Bali, Indonesia, by integrating wind speed data from MODIS and physical infrastructure parameters; they produced a map of the potential wind energy distribution and the amount of power that could be generated. To identify areas in Iran with the potential for wind power plants, Noorrollahi et al.^9^ conducted a case study that successfully integrated multiple criteria analysis methods involving physical and environmental aspects. Furthermore, several development-related studies have focused on modeling the potential of hydro-energy. Korkovelos^18^ compared two LISFLOOD hydrological models with their own model that used data from the Global Runoff Data Center (GRDC) and digital elevation model (DEM) observation stations. Additionally, Rojanamon^19^ conducted a review of the physical, environmental, and economic aspects of achieving hydropower energy development.

Some studies have modeled multiple energy sources to determine their combined potential for maximizing energy production.^17^ Wang et al.^20^ conducted spatial modeling of the potential for solar, wind, biomass, geothermal, and hydropower energy in Fukushima, Japan, resulting in a map of the combined renewable energy potential. Asadi and Pourhossein^21^ conducted research in eastern Azerbaijan using technical, economic, and environmental data to determine the areas suitable for the construction of solar and wind power plants. Moreover, Yeom et al.^22^ used the COMS MI satellite to determine the spatiotemporal distribution of potential solar and wind energy resources over North Korea. Other studies^23,24^ mapped hydro-solar-wind energy potential in West Africa and developed an optimally designed system of renewable energy resources based on the region of South and Central America. Previous studies have mapped multi-renewable energy sources by considering physical, social, and economic parameters. However, there has not yet been any research on this topic covering the Southeast Asian region as a whole and considering both a land suitability analysis and an estimate of the total potential energy produced.

There are many factors limiting the relevant research in this area. One reason is the changing dynamics of the weather, which can influence the available solar, wind, and hydro energy. Investigating weather changes either diurnally or seasonally is challenging because it requires observation tools with high temporal and spatial resolutions. For example to observe solar radiation and wind conditions which are location-dependent and greatly fluctuating, a high density of solar and wind observation sensors are necessary. Satellites equipped with a scatterometer to observe wind vectors, such as QuikSCAT (QSCAT), have low spatial and temporal resolutions; in the case of QSCAT, the spatial resolution is only 0.25° × 0.25° and the temporal resolution is only 3 days, making the satellite usable only in the ocean^25^. The new generation of satellite-scatterometers, named Advanced Scatterometers (ASCAT), has similar capabilities but with improved spatial resolution^26^. Therefore, it is necessary to develop studies for planning the optimal location for solar PV, windfarms, and hydropower stations that can support the efficient deployment of energy. In addition, it is necessary to develop the potential produced energy with consideration of various meteorological factors, which are all strongly influenced by weather conditions.

The limited number of observations has caused there to be no studies considering seasonal factors in modeling the optimum energy for solar, wind, and hydro energy. Therefore, this study aims to evaluate solar, wind, and hydro energy across the entire region of Southeast Asia. The objectives of the study included modeling the energy needs of urban and rural areas, determining the level of suitability for multi-renewable energy production, and calculating the total estimated energy potential of multi-renewable energy. The integration of dominant energy probabilities for solar, wind, and hydropower in urban and agricultural areas, which we believe to be the first attempt in the field, is also discussed. This study also creates a new model that integrates the energy requirements of the study area with its suitability for energy production, accompanied by an estimate of the amount of potential energy that can be generated. The results of this study are expected to be used by multi-level governments in the region as well as the private sector to inform energy and spatial planning policies and strategies to prioritize and accelerate the energy transition in Southeast Asia based on different regional characteristics.

## Results

### Spatial distribution of estimated urban and rural energy demand

**Figure 1:**
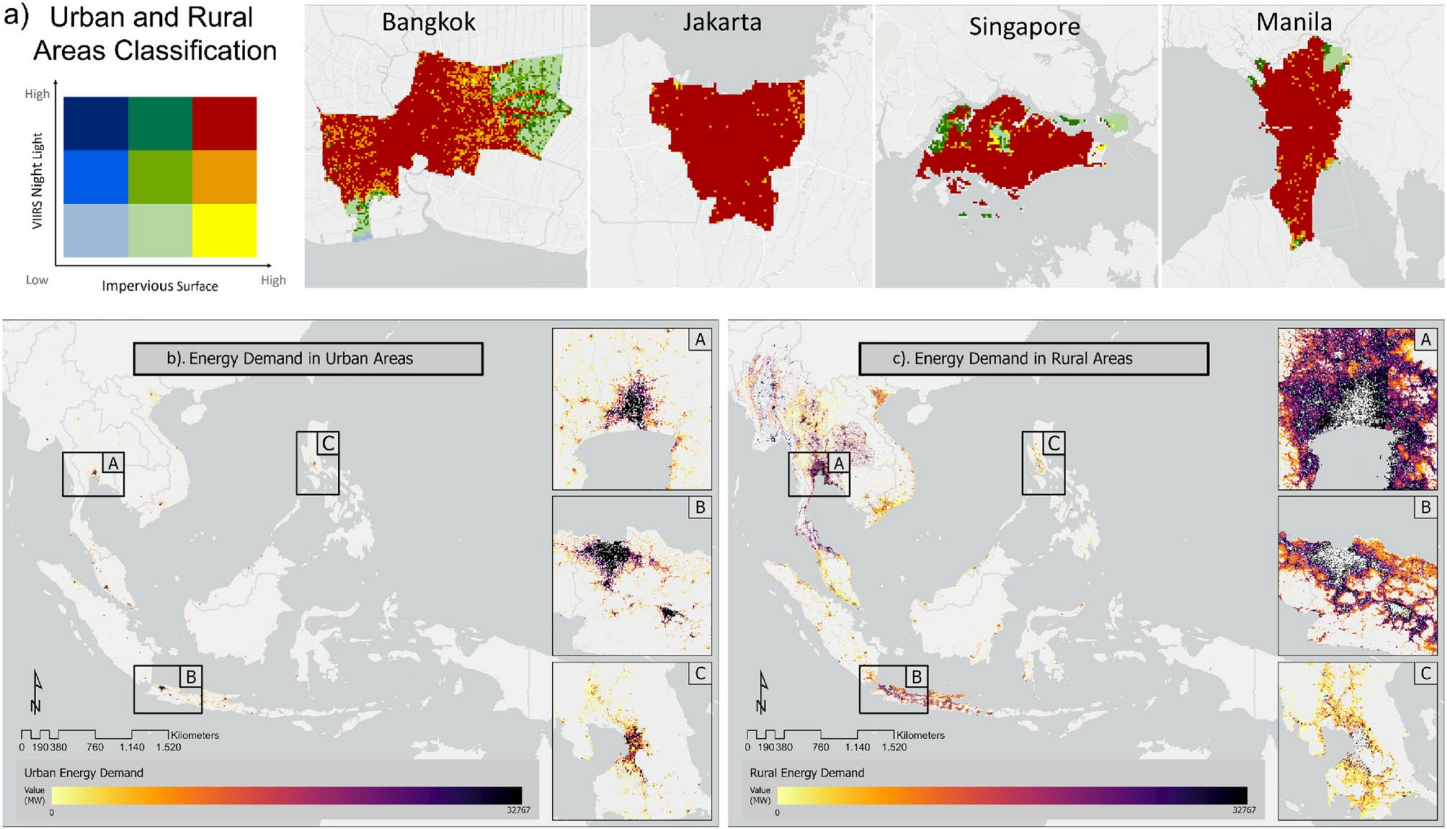
Urban rural area classification in four selected capital city in Southeast Asia (**a**) and total annual energy consumption in (**b**) urban and (**c**) rural areas. The insets represent the metropolitan areas of (**A**) Bangkok, Thailand, (**B**) Jakarta, Indonesia, and (**C**) Manila, Philippines.

In the analysis of the energy demand of Southeast Asia, the study area was divided into two land cover classes: urban and rural. In this study, the classification for these two land cover classes was created by combining nightlight and impervious surface data. Figure 1a depicts that the obtained land cover class can represent the urban class in selected four Southeast Asian capital cities. In addition, Table 1 shows the percentage distribution of urban and rural land cover classes in each of the six cities: Bangkok, Thailand; Hanoi, Vietnam; Jakarta, Indonesia; Kuala Lumpur, Malaysia; Manila, Philippines; and Singapore, Singapore. The cities were found to have the High (night light)–High (impervious surface) class as the majority, which was represented as urban area. The total consumption was calculated for a coverage area of 100 × 100 m in kilowatt-hours. Figure 1b,c show the distribution of electricity consumption in urban and rural areas in Southeast Asian countries. The dark shade indicates areas with more energy consumption; almost all urban areas with a high population density are displayed in red. The areas with low consumption are indicated in blue. The value of average electricity consumption per capita in urban areas was 10% higher than that in rural areas. The results showed that Indonesia had the highest total energy consumption, with a total electricity consumption of 212,747 GWh in 2020. The country with the lowest total electricity consumption was Timor Leste at 124 GWh. The comparative analysis of total electricity consumption between urban and rural areas showed that seven countries (Indonesia, Thailand, Vietnam, Myanmar, Brunei, Cambodia, and Laos) had higher total electricity consumption in rural areas compared to urban areas due to the higher proportion of rural areas. The areas around the capital cities Jakarta, Bangkok, and Manila were selected to approximate the energy consumption in the urban centers.

**Table 1:** Percentage of rural–urban classification in six capitals cities of Southeast Asia countries.

City	L–L (%)	L–M (%)	L–H (%)	M–L (%)	M–M (%)	M–H (%)	H–L (%)	H–M (%)	H–H (%)	Area (km^2^)
Bangkok	0.54	0.03	0.00	13.36	5.46	4.27	1.11	14.40	60.83	1626
Hanoi	0.00	0.00	0.00	1.76	0.59	3.72	3.72	6.85	83.37	127
Jakarta	0.26	0.00	0.00	0.37	0.00	0.04	0.30	3.43	95.61	671
Kuala Lumpur	0.00	0.00	0.00	0.00	0.00	0.00	1.23	4.63	94.14	243
Manila	0.04	0.00	0.00	4.09	0.81	2.51	0.26	5.71	86.57	586
Singapore	0.00	0.00	0.00	6.67	1.45	6.12	2.35	2.75	80.66	637

With L = Low, M = Medium, H = High.

### Suitability index of multi-renewable energy

**Figure 2:**
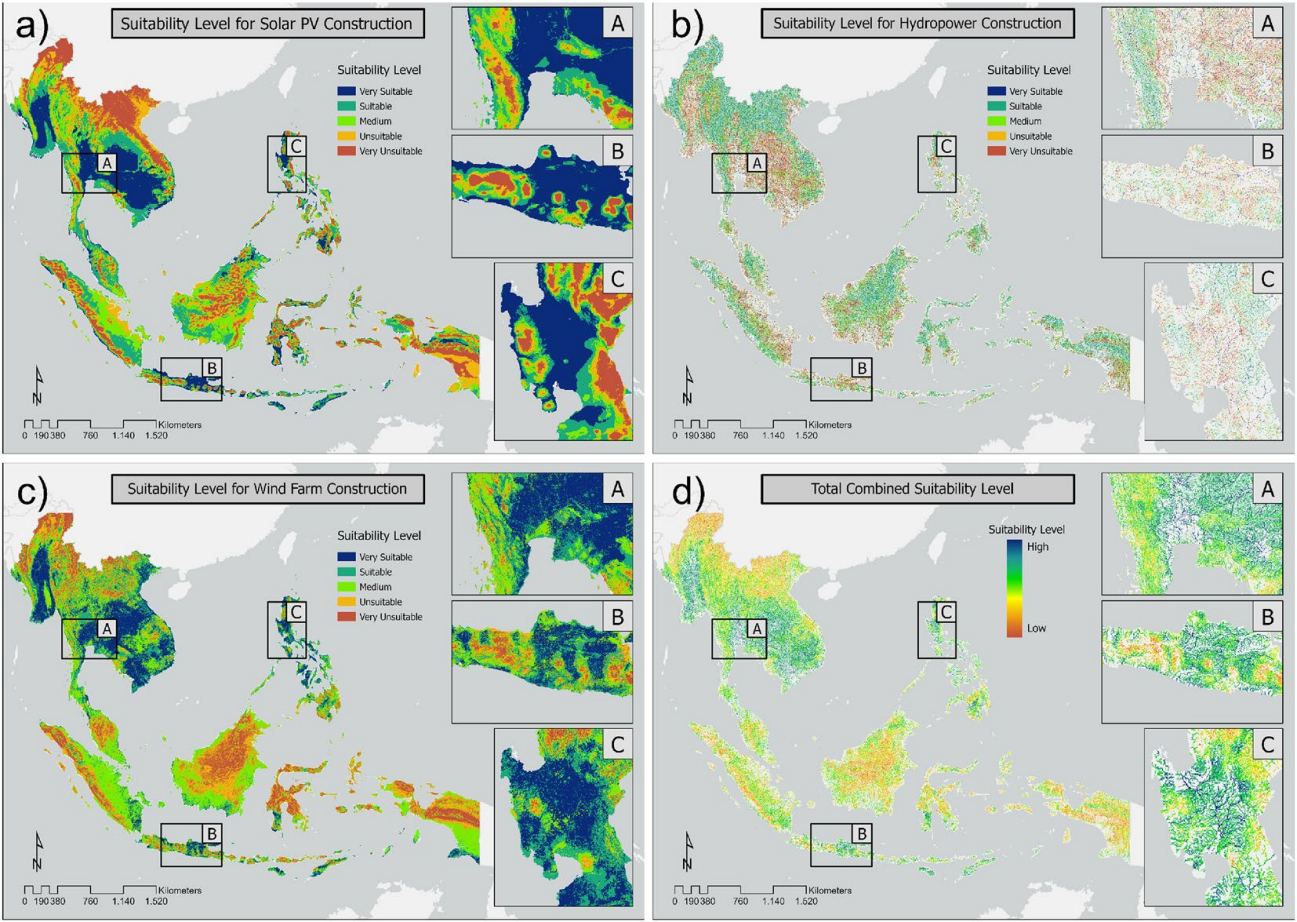
Levels of suitability for developing (**a**) solar photovoltaic (PV) energy, (**b**) hydropower, and (**c**) wind power infrastructure in Southeast Asia, and (**d**) the total combined suitability levels for all three types of power.

The suitability of areas for the development of solar, wind, and hydropower energy infrastructure were classified at five levels: very suitable, suitable, medium, unsuitable, and very unsuitable, with a spatial resolution of 1 km. Figure 2a shows the results of the land suitability analysis for solar PV development in Southeast Asia. An area of 40,923,000 ha was determined to be very suitable for solar PV construction. Indonesia has the highest potential area, accounting for 23.66% of the total land area in the “very suitable” and “suitable” categories (approximately 35,681,200 ha). Even though Indonesia had tremendous solar PV construction potential, its potential was no more significant than that of Thailand when only the “very suitable” category was considered. One of the reasons for this is that Indonesia is often covered by clouds, thus affecting the amount of Global Horizontal Irradiance (GHI) that reaches the Earth’s surface.

The suitability levels for wind energy development in Southeast Asia are shown in Fig. 2b. Some areas with a high level of suitability included the north-eastern region of Thailand, which is dominated by relatively flat agricultural land, and the islands of Sumba and Timor near the equator, where the geography is mostly vast vacant land with a gentle slope. Despite the Luzon and Visayas areas in the Philippines being relatively densely populated, their geographical conditions are flat and supported by good infrastructure access. Figure 2c shows the suitability level of hydropower energy for rivers in Southeast Asia. Generally, rivers with a high stream order had reasonably high suitability index values. In addition, river networks in mountainous and rural areas had reasonably high suitability index values compared to areas near the coast and urban regions. Indonesia had the most significant potential of all the studied countries, with approximately 493 km^2^ or 35.94% of its area being considered suitable or very suitable. The second most suitable country was Myanmar, which had an identified suitable area of 298 km^2^ (21.71%), followed by Laos and Vietnam, with suitable areas of 124 km^2^ (10.03%) and 117 km^2^ (9.16%), respectively. An integration process was carried out on the three suitability index results to determine the areas with the highest and lowest overall renewable energy suitability. Figure 2d shows the results of the overall suitability index analysis.

### Solar, wind, and hydropower energy potential

**Figure 3:**
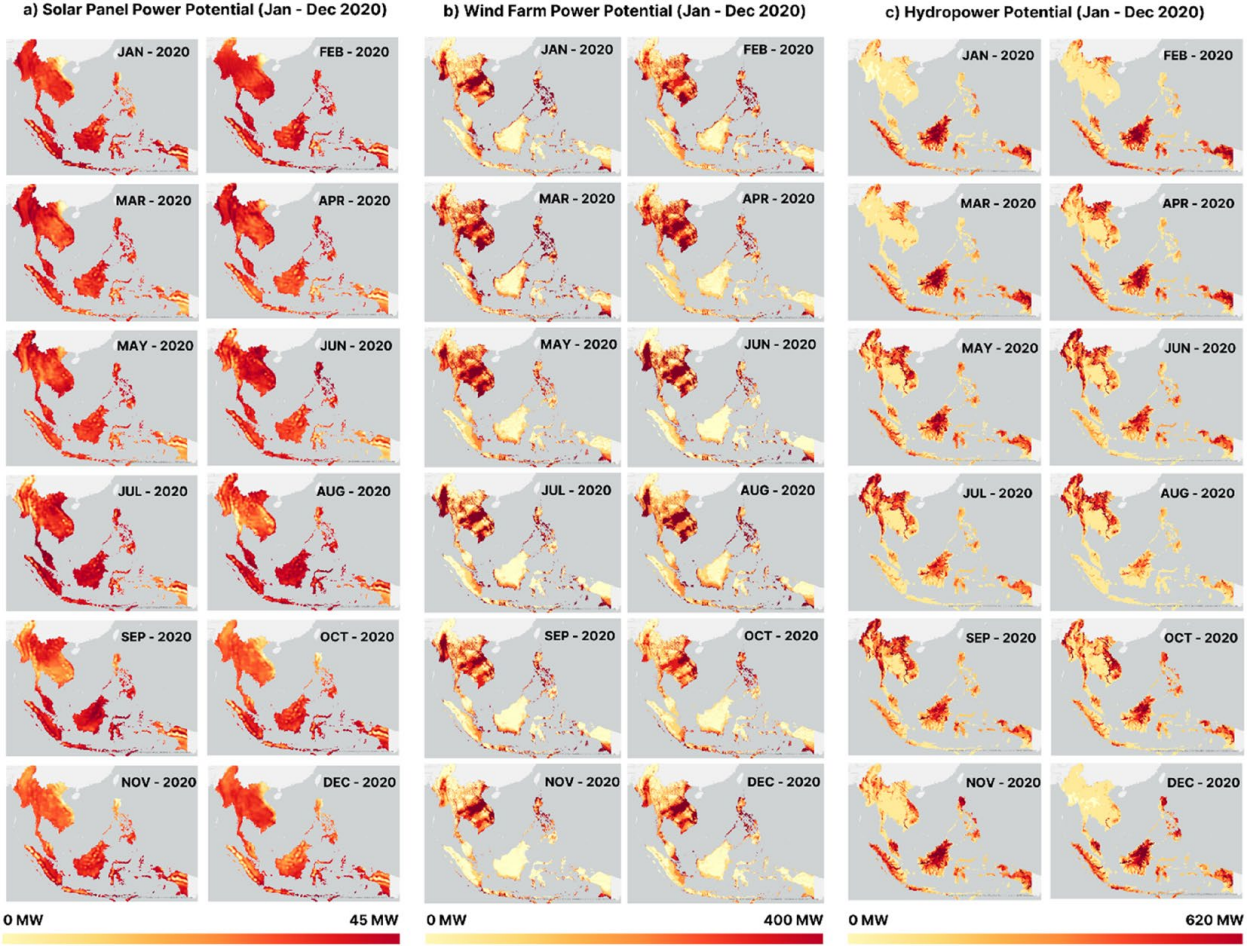
Map visualization of monthly power potential (January–December 2020) for (**a**) solar panels, (**b**) wind farms, and (**c**) hydropower.

The potential for adequate solar, wind, and hydropower over 12 months in Southeast Asia is shown in Fig. 3. Figure 3a shows the effective power potential of Southeast Asia's solar panels, which was calculated by combining the conditions of solar irradiance and the effects of temperature, aerosol optical depth (AOD), and rain. The highest energy potential produced by solar panels occurred in October (21.386 GWp), whereas the lowest was in April (2.087 GWp). Figure 3b shows the wind energy potential for 12 months in Southeast Asia. The generated power varied each month and was mainly influenced by wind speed. The maximum power generated was 400 W/m^2^ in the highest class, assuming a wind height of 100 m. The processing results showed that the highest estimated power production occurred from June to August, whereas April and October had the lowest energy production. The estimated monthly energy produced from hydropower in Southeast Asia countries is shown in Fig. 3c. The figure shows the monthly pattern of estimated hydropower potential in the 12 month period of 2020. It can be seen that there are two different patterns in the northern countries of Southeast Asia, such as Myanmar, Vietnam, Thailand, Laos, Philippines, and Cambodia, compared to the southern parts, such as Indonesia, Malaysia, Brunei, Singapore, and Timor Leste.

**Figure 4:**
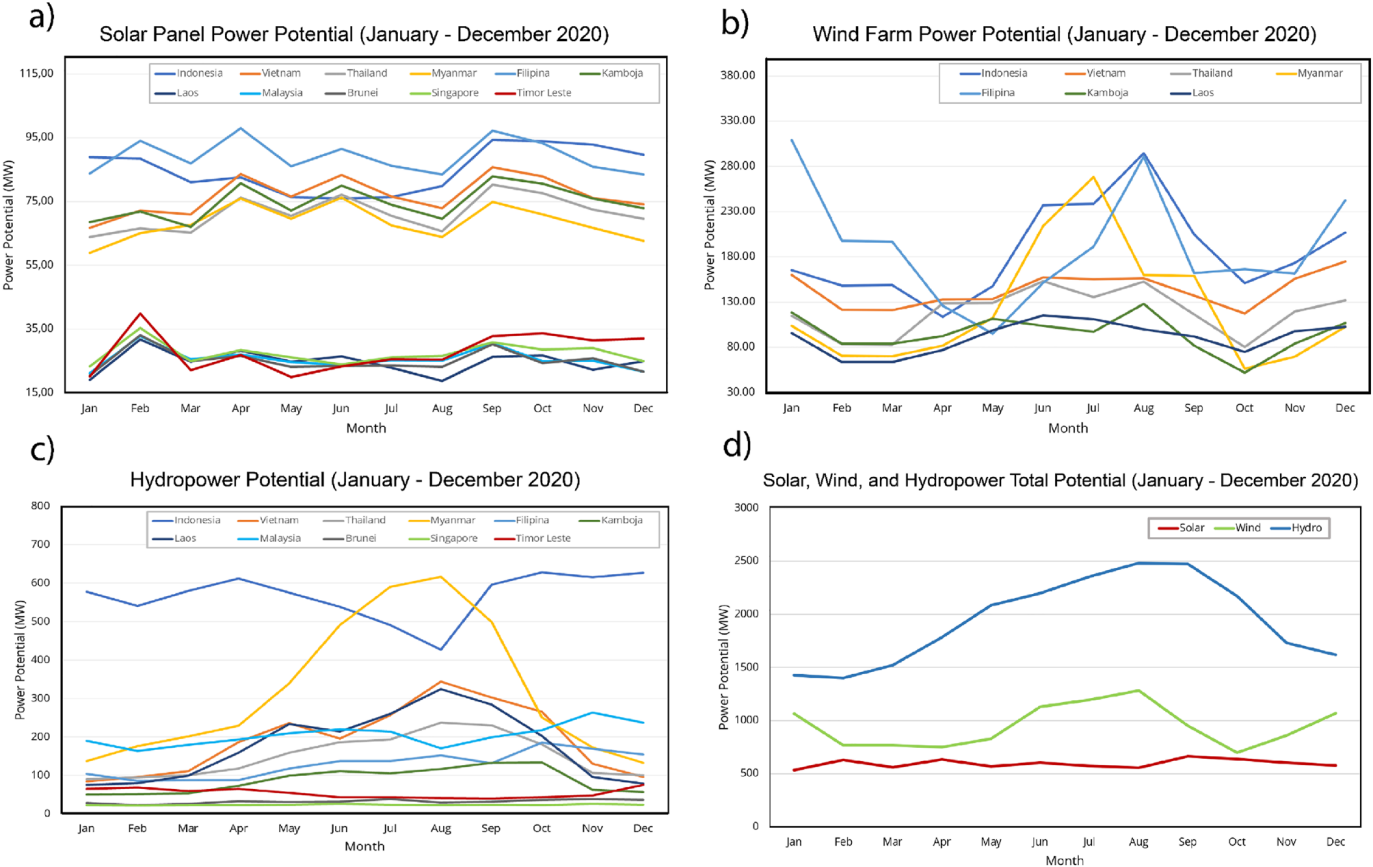
Country profile of monthly power potential from January to December 2020 for (**a**) solar photovoltaic (PV) power, (**b**) wind farms, (**c**) hydropower, and (**d**) the combined total for solar, wind, and hydropower in Southeast Asia.

A 12 months time-series graph of the potential solar, wind and hydro power in 11 countries in Southeast Asia is shown in Fig. 4. The countries with the highest potential solar PV power are Indonesia, the Philippines, Vietnam, Cambodia, Thailand, and Myanmar (Fig. 4a). For Indonesia, the graph shows that April to August had a downward trend with a considerable potential for solar panel power (approximately 70 MW), and the trend rose in September to reach the highest potential (94.346 MW). Vietnam, Cambodia, Thailand, and Myanmar showed nearly identical trends, where the lowest potential (approximately 60 MW) occurred in January, and the potential then increased from February to March. These four countries reached enormous power potential, with Vietnam reaching 85.779 MW, Cambodia reaching 82.879 MW, Thailand reaching 80.263 MW, and Myanmar reaching 75 MW. Countries with low potential were Timor Leste, Singapore, Laos, Malaysia, and Brunei. The trend in Timor Leste decreased and then increased, with the potential in January being 20 MW, rising to the highest potential of 39.924 MW in February, and then decreasing again in March to 22 MW. Singapore, Laos, Malaysia, and Brunei showed nearly the same trends. They had the lowest potential in January, then stabilized, decreased until August, and rose again in September.

Figure 4b shows a monthly time-series graph for seven countries based on the power potential analysis for wind farms. Similar to the solar PV power potential, these values were taken from the average of all rasters per country. The seven countries experienced peak power from July to August. The power then decreased slowly and tended to stabilize until April, and then began to rise again in May. The Philippines was the country with the highest initial power potential, with 308 MW in January. The power then decreased moderately to approximately 95 MW in May before rising slightly and reaching a peak of 291 MW in August. From this point forward, the potential power of the Philippines experienced a decline and remained stable until November, before experiencing another increase in December. Regarding the other countries with less initial generated power potential, Indonesia, Vietnam, Thailand, Myanmar, Cambodia, and Laos started the year at around 165 MW, 160 MW, 115 MW, 103 MW, 118 MW, and 96 MW, respectively. The graphs remained stable until April, then climbed steadily and peaked at just above 290 MW in August for Indonesia, 268 MW in July for Myanmar, and 152 MW for Thailand. The power of Vietnam, Cambodia, and Laos was relatively stable from February to April before experiencing a steady increase and then decrease from July to August.

Figure 4c shows the monthly time-series graph of the hydropower potential. In the period of June–August, countries in southern Southeast Asia experienced a decline, where August experienced the minimum of the potential hydropower value. Meanwhile, the opposite pattern was seen for countries in the north; from May to September, there was an increase, with the peak occurring in August. This unique pattern occurred due to differences in the rainfall phenomena that occur in Southeast Asia, which are not uniform for the eleven countries over each month. Indonesia is the leading country in terms of hydropower potential, where the peak reached 626 MW and the lowest value was 426 MW. The countries that have relatively low hydropower potential values are Singapore, Brunei, and Timor Leste, whose largest potential values are 26 MW, 38 MW, and 75 MW, respectively. Figure 4d shows the solar, wind, and hydropower accumulation total for the combination of all regions in Southeast Asia. Hydro has highest total accumulative value compared with wind and solar. August and September are the months where hydropower reaches its maximum energy potential, while wind power is highest in August and January. Cumulative solar PV power is relatively constant throughout the year and exhibits the lowest power potential compared to other renewable energy sources.

**Figure 5:**
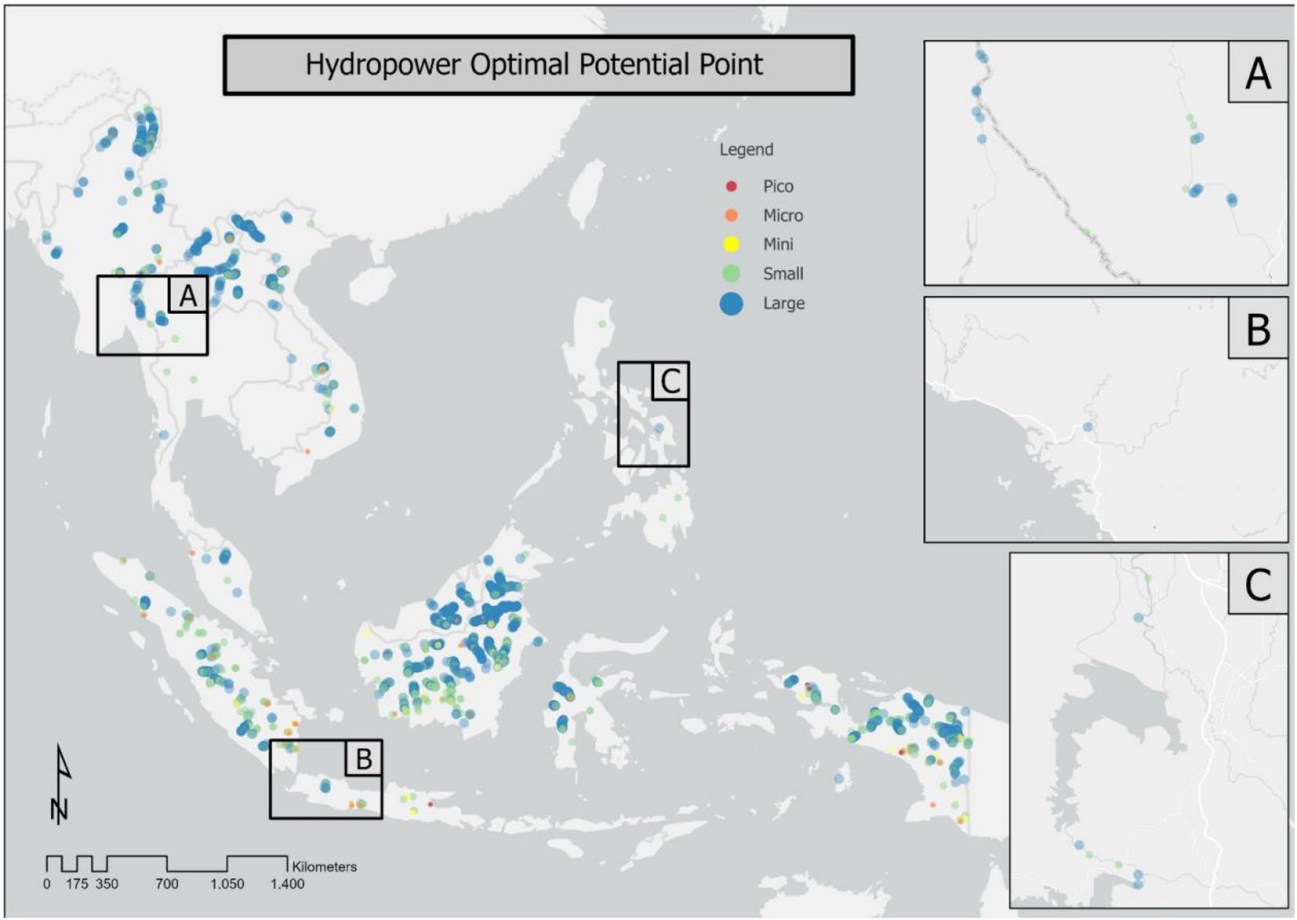
Potential optimal points for hydropower potential in Southeast Asia.

The combined results showed potential points for power plants of various scales. Figure 5 shows that hydropower plants on a small scale (micro, with an energy potential of 5–100 kW; mini, with an energy potential of 100–1000 kW; small, with an energy potential of 1000–25,000 kW; medium, with an energy potential of 25,000–100,000 kW; and large, with an energy potential of > 100,000 kW^27^) could be located in rivers with minor flow accumulation and stream order compared to medium- to large-scale power plants. Upstream rivers would be dominated by micro- and mini-scale power generation classes. In contrast, larger downstream rivers, such as the Mekong, which flows across Myanmar, Laos, Thailand, Cambodia, and Vietnam, would be dominated by medium and large power plants. Other large rivers, such as the Pahang River in Malaysia and the Indragiri River in Indonesia, are also included in the small and medium classes (micro, mini, small). Such rivers can have this designation because rivers with significant flow accumulation and stream order have a relatively large discharge value. However, with regard to the head parameter, upstream rivers have more potential because of their steep slopes. For all of the types of hydropower plants combined, Indonesia had 1897 possible optimal points, which was the highest number compared to the other countries (56.25% of all points). Cambodia had the next most potential optimal points with 345 (10.23%), then Myanmar with 329 (9.75%), and Laos with 245 (7.26%).

**Figure 6:**
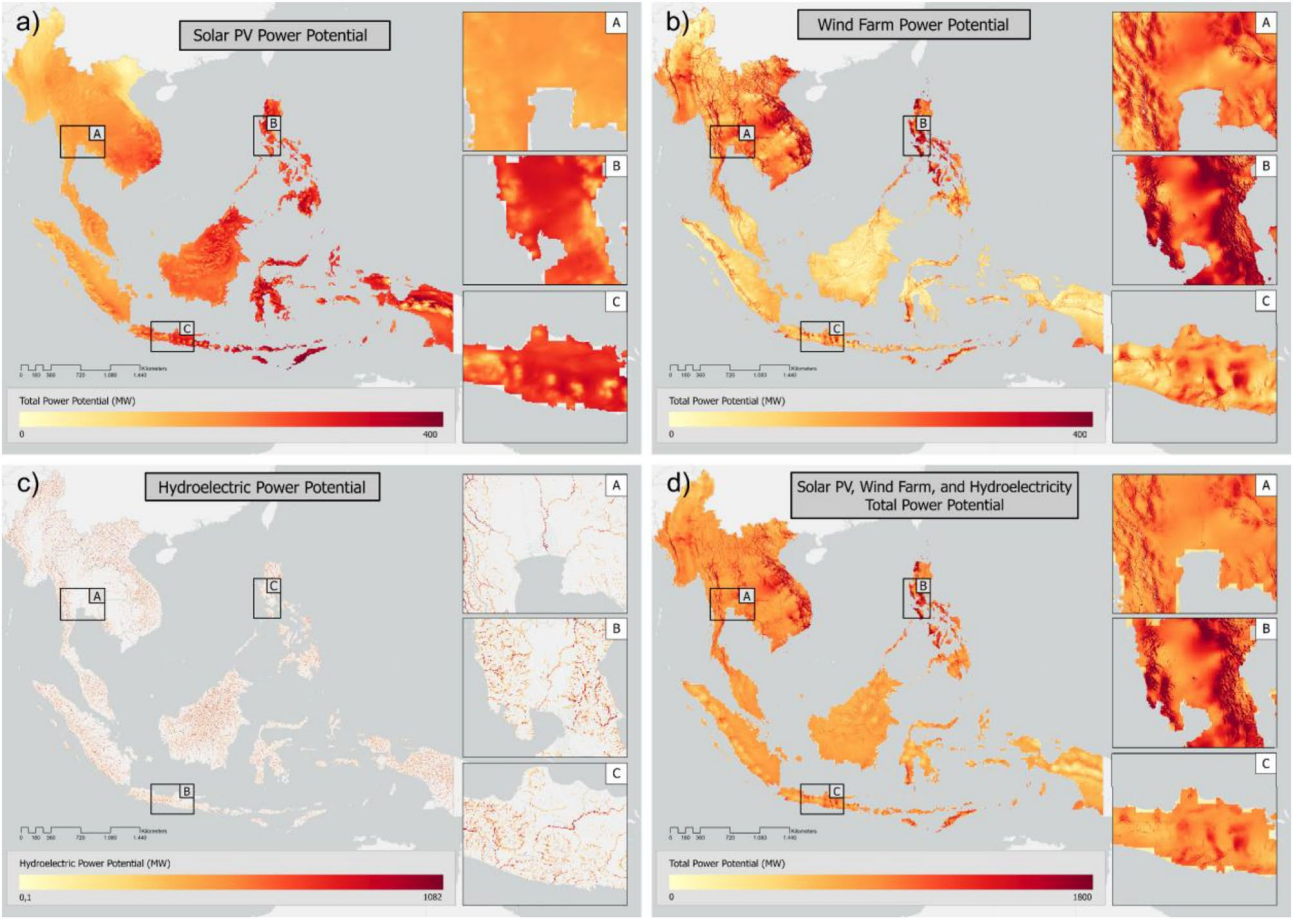
Power potential for (**a**) solar photovoltaic (PV), (**b**) wind, and (**c**) hydroelectric power, as well as the (**d**) total combination of the three types of power.

The annual generated power potential for multi-renewable energy construction of solar (Fig. 6a), wind (Fig. 6b), and hydropower (Fig. 6c) plants in Southeast Asia was also analyzed. The distribution of the potential energy value had different characteristics for each type of power plant. Therefore, a combined analysis of the power potential generated from the three sources of renewable energy was carried out to determine the total power potential in each area of Southeast Asia. Based on Fig. 6d, the areas with the highest combined power potential are mainly in northern Southeast Asia, including parts of Vietnam, Laos, Thailand, and the Philippines. Areas close to the equator, such as Indonesia, Malaysia, Brunei, and Singapore, have a lower potential than northern countries, except for southern West Nusa Tenggara (Indonesia) and Timor Leste. These results occurred because the potential for wind power is enormous in the northern area of the region, which leads to considerable differences in the combined power of the three energy sources.

### Multi-renewable energy in specific urban, rural, and agricultural areas

**Figure 7:**
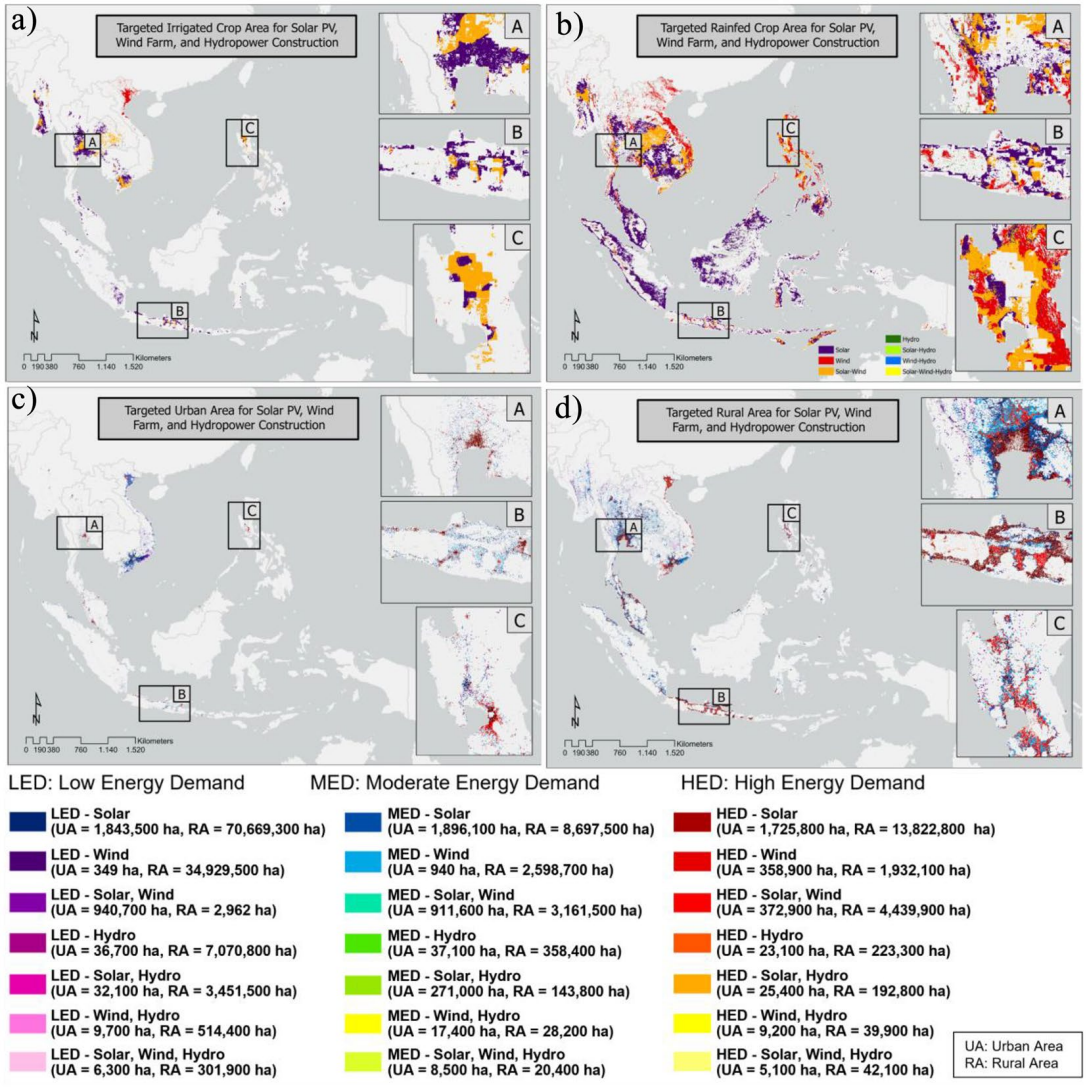
Demands for multi-renewable energy in (**a**) irrigated cropland, (**b**) rainfed cropland, (**c**) urban areas, and (**d**) rural areas in Southeast Asia. The total area of the three groups was calculated both for urban and rural areas.

Figure 7 shows the dominance analysis of multi-renewable energy based on the potential power generated by solar panels, wind farms, and hydropower. This analysis was carried out by classifying the potential power into five classes and considering only the class with the greatest potential. The highest classes were then combined to determine the dominant areas for the construction of solar PV, wind, and hydropower plants, as well as their combinations. Overall, solar PV plant construction is the most area-intensive type of energy generation among the considered energy sources, requiring 143,901,600 ha (61.71%), followed by wind (39,618,300 ha; 16.98%); a combination of solar PV and wind (37,302,500 ha; 16%); hydro (7,665,200 ha; 3.28%); a combination of hydro and solar PV (3,792,500 ha; 1.62%); and a combination of hydro and wind (582,700 ha; 0.25%). Most of Indonesia's territory is dominated by potential energy production via diesel, as are northern regions including Laos, Cambodia, and Thailand.

There is ample wind potential in northern regions, such as the Philippines, Vietnam, Laos, and Thailand, and a combination of solar PV and wind farms is also dominant in these areas. For the three types of renewable energy combined, the areas with the greatest potential are parts of Vietnam and Laos, as well as the northern Philippines. In this study, the analysis of the dominance of multi-renewable energy was carried out for four specific target areas, namely irrigated agriculture, rainfed agriculture, urban areas, and rural areas, to identify the areas with the most potential for constructing renewable energy. The urban and rural areas were divided into three classes of energy demand: high (HED), moderate (MED), and low (LED). This was done by classifying the estimated urban and rural energy demand (Fig. 1) into three categories using the geometric interval method, and then overlaying these estimations with the three types of renewable energy. This classification process details the energy potential based on the characteristics of each region. An analysis of the agricultural area was conducted to determine the potential of renewable energy in supporting sustainable food security. For example, the water pumps used to irrigate agricultural areas are expected to be powered by renewable energy rather than fossil fuels in order to increase agricultural productivity. In urban areas, the massive energy demand can be addressed by utilizing environmentally friendly energy sources currently receiving public attention, such as through installing solar panels on the roofs of buildings. In urban and rural areas, it is possible to use the construction of solar panels, wind farms, and hydropower to achieve energy distribution, especially in areas that do not yet have electricity.

Figure 7a, b show the solar, wind, and hydroelectric energy demands for agricultural areas with irrigated and rain-fed crops. The rainfed area in Southeast Asia is larger than the irrigated crop area, meaning that rain-fed agricultural systems are dominant. Additionally, in rainfed crop areas, energy sources can also be used to assist pumping and irrigation processes, so that they do not rely solely on rain for irrigation. Irrigated agricultural land is dominated by solar and wind power potential. The areas with the highest probabilities of combined renewable energy are southern Myanmar, Thailand, and Vietnam. In the rainfed agricultural area, the potential for these three types of energy is equal. Most of Indonesia's territory and northern countries such as Myanmar, Laos, Cambodia, Vietnam, and the Philippines are covered by areas with solar and hydro potential. There is also ample wind potential in northern countries, such as the Philippines, Vietnam, Laos, and Thailand, and it is possible that solar panels and wind farms could be combined in these areas. It is most likely for the three energies to be combined in the rainfed agricultural regions of Vietnam and Laos, as well as in the northern and southern parts of the Philippines. Figure 7c,d show the urban and rural areas for which the construction of solar panels, wind farms, and hydroelectric power plants could be targeted. Urban areas with the highest energy demand have the most solar potential, including Bangkok (Thailand), Hanoi (Vietnam), Manila (Philippines), and Jakarta (Indonesia).

It is recognised that the transition to renewable energies such as solar energy will intensify the global competition for land and drive land use change, resulting in further environmental impacts. Focusing on the EU, India, Japan, and South Korea, Van de Ven et al.^28^ found that solar energy may occupy as much as 5% of the total land area to produce 80% of the electricity in these areas by 2050. This land requirement is significant; for illustration, 4% of the land in the EU land is currently covered with manmade surfaces^29^. A solution to this problem is to integrate solar energy into urban spaces, for example, by placing small-scale PV systems on the roofs of buildings, even though the potential is somewhat limited; only around 3% of urbanized surfaces are suitable for such purposes with reasonable efficiences^30^. Urban areas typically have high energy needs^31^, and most of these areas are covered by solar potential. Solar PV installation on the rooftops of buildings has the potential to provide clean energy sources in urban areas. The deployment of rooftop solar PV has been increasing in recent years, especially with the increased interest in the net-zero energy building (NZEB) concept^32^. To achieve NZEB goals, buildings are expected to install sustainable energy sources such as rooftop PV systems, especially for commercial and high-end residential buildings^33,34^.

In rural areas with high energy demands, the three types of renewable energy are equally dominant. This is because constructing wind turbines with heights of 100 m requires a large area, and hydropower plants are constructed in existing river networks that are primarily found in rural areas. Rural areas with high energy demand due to a high concentration of industry are mainly found in Java (Indonesia), Brunei, Malaysia, southern Thailand, and northern Vietnam. In this analysis, three samples were taken from urban and rural areas based on the prominent differences in their energy demands and renewable energy potential. The three urban samples highlighted, namely Bangkok (Thailand), East Java (Indonesia), and Manila (Philippines), each have characteristics that are intermediate between urban and rural areas. Urban areas have high energy needs, and most of these areas are covered by solar potential.

Table 2 lists the area distribution for the potential of each type of energy and its combination in eight targeted areas. Physical characteristics and the energy demand are important factors that determine the type of energy that can support each targeted area. Overall, it can be seen that solar energy dominates the urban, rural, and agricultural regions. Urban areas that already have energy infrastructure based on fossil fuels are challenged with an energy crisis. Therefore, it is time to transition to alternative energy sources, and this can be achieved because, although the energy demand in urban areas is increasing, the supply of energy sources, such as solar power, is also very high. In addition to the potential of solar panels, the potential for wind power development is also high in MED and LED urban areas. With a large supply of energy sources, rural areas should be able to use energy that is not as widespread as in urban areas. The energy demand in rural areas is high, but there are still residential areas that do not have electricity. Rural areas therefore exhibit unique patterns based on their energy needs. The lower the energy demand in the area, which corresponds to lower population levels, the greater the potential for building wind and hydropower plants. This is especially the case considering that such facilities require a large land area and, in the case of wind farms, must be far from settlements, or in the case of hydropower plants, require a network of rivers with large discharges that can generate considerable power. As previously mentioned, an analysis of the agricultural area was conducted to determine the potential of renewable energy in supporting sustainable food security. The amount of diesel fuel used in agricultural areas supports the currently developing technology of agri-voltaic power, in which photovoltaic panels are placed above crops to increase agricultural productivity while reducing environmental impacts^35^. The likelihood of developing hydro and wind farms is greater in rain-fed agricultural areas than in irrigated areas. The three renewable energy sources involved in this study require suitable locations to produce large potential power generation and specific regions for their optimal utilization.

**Table 2:** Dominant area (ha) for each type of renewable energy in each targeted area.

Targeted area	Dominant renewable energy in the targeted area (ha)						
	S	W	H	SW	SH	WH	SWH
Urban-HED	1,725,800	358.900	23,100	372,900	25,400	9200	5100
Urban-MED	1,896,100	940	37,100	911,600	271,000	17,400	8500
Urban-LED	1,843,500	349	36,700	940,700	32,100	9700	6300
Rural-HED	13,822,800	1,932,100	223,300	4,439,900	192,800	39,900	42,100
Rural-MED	8,697,500	2,598,700	358,400	3,161,500	143,800	28,200	20,400
Rural-LED	70,669,300	34,929,500	7,070,800	2,962	3,451,500	514,400	301,900
Agri-irrigated	157,940	27,256	2,250	70,111	1,367	581	505
Agri-rainfed	827,887	223,361	33,182	254,553	20,206	3454	2778

*HED* high energy demand, *MED* medium energy demand, *LED* low energy demand, *S* solar power, *W* wind power, *H* hydropower.

## Discussion

**Figure 8:**
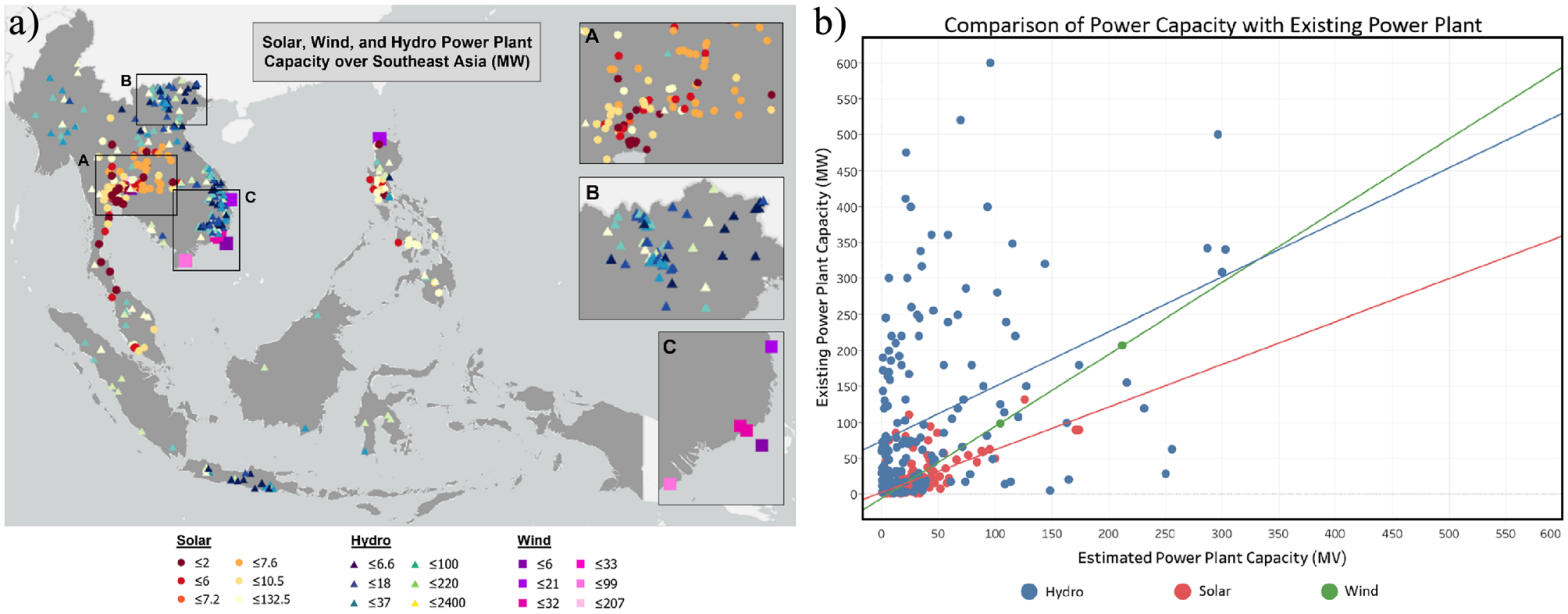
(**a**) The distribution of solar, wind, and hydropower plants in Southeast Asia and their generating capacity. (**b**) Comparison of the estimated power capacity of solar, wind, and hydropower plants with the existing power plants described in the Global Power Plant Database^36^.

Figure 8A shows the distribution of solar, wind, and hydropower plants in Southeast Asia and their generating capacity. There are 246 solar power plants, 7 wind power plants, and 214 hydropower plants that were compared using the root mean square error (RMSE) and R^2^. Figure 8b shows a scatter plot of the results of the comparison between our research products and the data from the Global Power Plant Database in the form of estimated power generation. The R^2^ value for the comparison of estimated and actual solar energy was 0.8323, with estimated solar panel power tending to be overestimated compared to the actual power capacity. This value can be influenced by parameters not included in the calculation, such as cloud cover, solar PV tilt azimuth angle, and solar PV working temperature. In addition, the efficiency of the solar cells was defined as 15% assuming that the solar cells were made of crystalline silicon, which is not always the case for solar power plants in Southeast Asia. The R^2^ value for comparing hydropower energy was 0.9986, indicating that the estimated and actual datasets had a reasonably strong relationship. The resulting RMSE value was significant, with a value of 6.39. This value may have been influenced by the assumption used in this study that the adequate working time of wind turbines is 24 h; therefore, the potential theoretical energy was overestimated. In addition, the sensitivity of the analysis was affected by the limited number of power plants built in the study area. Any significant change in the data affects the overall results. The R^2^ value for the comparison of estimated and actual wind energy was 0.4679, and the estimated power value for hydropower tended to be underestimated. This condition is the opposite of that for solar panel power in that the data with low values had considerable differences compared to the data with higher values. This shows that the results of the power calculation in this study had a higher sensitivity for medium- and large-scale power plants. In contrast, small-scale power plants would need to be further developed so that the study results would be more sensitive. Improved sensitivity could also be achieved by using additional parameters, such as the length of the diversion path, and a more precise definition of turbine and generator efficiency figures for small-scale power plants.

**Figure 9:**
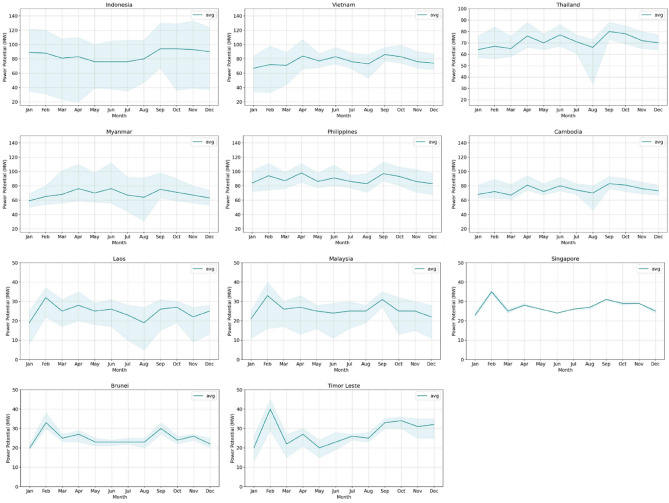
Time series analysis of the sensitivity of potential solar photovoltaic (PV) energy in Southeast Asian countries.

The estimates for solar energy in comparison with the data in the power plant database had the lowest RMSE values of all the energy types. Therefore, this study attempted to specifically determine the monthly fluctuating sensitivity levels of solar energy in each Southeast Asian country. The sensitivity range was obtained by comparing each month's average, minimum, and maximum potential power values; the wider the range, the lower the sensitivity level. Figure 9 shows the trend for each country’s solar power potential sensitivity level. The varying levels of sensitivity in each Southeast Asian country are influenced by several factors, including the size of the country's administrative area and geographical conditions. Indonesia has the most extensive range of potential values among all other Southeast Asian countries; this means that Indonesia's sensitivity level for its potential power was relatively low. In contrast, Singapore and Timor Leste had the smallest value ranges. These results indicate that the monthly power potential in Singapore and Timor Leste have relatively high sensitivity. The broad range of sensitivity values for Indonesia is influenced by the country’s large area and its diverse and vast geographical landscape. In contrast, Singapore and Timor Leste are relatively small in area and have relatively homogeneous geographical landscapes. The highest average potential power values (~ 100 MW) were observed in the Philippines in February, April, and September. The same pattern was observed for Laos, Malaysia, Singapore, Brunei, and Timor Leste. There was a significant increase in the potential power in February and a decrease from June to August. This result indicates that seasonal and weather changes affect the potential power values of these countries. In the dry season, the power generated by solar PV is much greater than that generated in the rainy season. The monthly average value of the potential power in Indonesia is relatively stable (80–98 MW) each year. However, some areas have high rainfall in the dry season due to various geographical and topographical conditions; therefore, each region does not have the same characteristics and patterns of potential generated power.

The results of the comparison between generated energy and actual conditions using the Global Power Plant Dataset revealed that the hydropower calculation results were underestimated for large hydropower plants, but quite accurate for small hydropower plants. For example, the San Roque Hydropower Plant in the Philippines has a capacity of 411 MW but is estimated to be only 21 MW, and the Asahan 1 plant in Indonesia has a capacity of 186 MW but is estimated to be only 78.9 MW. In terms of hydropower plants that can be accurately estimated, Yan-Tann-Sien (Mini) in Vietnam has an actual capacity of 0.30 MW and an estimated capacity of 0.33 MW; Bersia (Medium) in Malaysia has an actual capacity of 48 MW and an estimated capacity of 46.82 MW; and Tarpein-1 (Large) in Myanmar has a capacity of 240 MW and an estimated capacity of 231.5 MW (Fig. 8).

Despite the optimistic future for Southeast Asia’s shift to renewable energy as well as its projected economic boost with significant macroeconomic attainment, this region still faces great challenges in terms of policy and regulation. To achieve the target of a 23% energy mix sourced from renewable energy in 2025, governments in Southeast Asia must build more renewable energy-based power plants. This study will help in the crucial phase of determining the feasibility and viability of such projects; it is a so-called prefeasibility study, in which potential sites are identified, short-listed, and studied in detail. This study can be a source for technical ministries and local government in terms of authorization and the application of licenses and/or permits. Governments and state-owned enterprises are among the important stakeholders for the adoption of relevant energy policies. After all legal, administrative, and business requirements have been met, the construction and operational phase will follow. Thus, the target supply of renewable energy can be easily fulfilled in the future.

This study had some limitations primarily due to data availability. The analysis of urban and rural energy demand involved limited parameters, such as the defining of urban and rural areas based on population density, impervious surfaces, and nightlight parameters. Furthermore, electricity consumption data are only available at the state level. These factors resulted in difficulties in identifying priority areas and effects for determining LED, MED, and HED in the analysis of specific target areas. For solar power specifically, the solar radiation data that were used had a spatial resolution of 20 km, whereas the results in this study were presented at 1 km. It is therefore recommended to use solar radiation data with higher resolution, such as that from MODIS, Himawari, or very-high resolution satellites. The inclusion of several additional parameters, such as the temperature of the solar panel and the tilt of the panel during operation, could also improve the quality of the power value to bring it closer to the actual conditions. Furthermore, the analysis of wind energy production can be further developed by integrating additional parameters, such as soil conditions, plate activity, and tidal riding, to analyze the suitability of the location of wind energy generators. For hydropower site analysis, several additional influential parameters of river characteristics should be considered, such as the hydrology cycle, suspended sediment, and irrigation networks. The temporal resolution can also be increased to consider more than the average river discharge for 1 year. It is necessary to use a higher basin level to analyze the effect of rainfall on the hydropower generation potential. Energy grid transmission data could also be used to help analyze the priorities for deployment of renewable energy sources. However, the priority development level necessitates further research to develop the methods for priority analysis, which must consider various aspects of accessibility, cost, and investment; to calculate the risk of vulnerability to disasters; and to calculate the air pollution factor and ultimately allow the analysis of the contribution of renewable energy to carbon neutralization. We also recommend that future studies incorporate climate model parameters; for example, they could account for the influence of El Niño and La Niña on solar radiation, wind speed, and the resulting river and basin discharges. For comparative analyses, better validation and verification methods are required, for example, by conducting direct measurements in the field.

In this study, research was conducted to determine the potential of multi-renewable energy sources in 2020. This year was chosen as the research period based on the novelty of the data and differences in topographical phenomena that occurred due to the COVID-19 pandemic. By conducting research in 2020, these results can be a starting point for a new pattern due to natural changes that occurred to allow for predictions in the next year after COVID-19. One limitation of this study is that it does not provide a predictive model, so the results are most accurate in representing the year 2020. However, this does not mean that these results cannot be used for subsequent years because topographic and meteorological conditions will not change significantly in a short period of time. In addition, the results of this study can be used in the long term projection and assist in creating predictions by adjusting the Representative Concentration Pathway (RCP) climate scenarios adopted by the IPCC^37^, one of which describes changes in meteorological conditions in different scenarios in the next decade. Currently these pathways are combined with Shared Socioeconomic Pathways (SSPs)^38,39^, which are scenarios of projected socioeconomic global changes under different climate policies.

## Methodology

**Figure 10:**
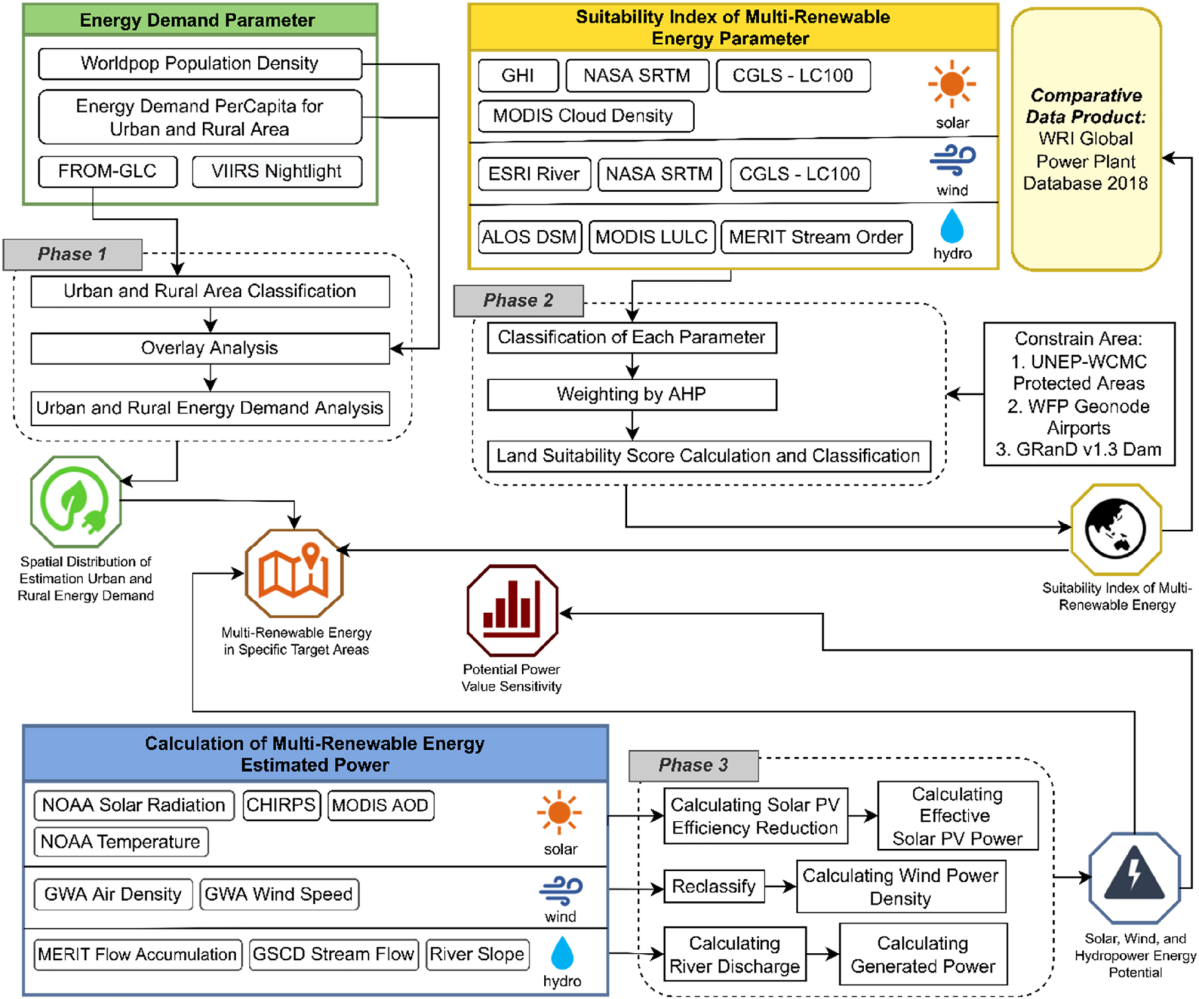
Flowchart of the methodology used in this study.

Data processing was divided into three phases. The first phase included the estimation of the energy demand in urban and rural areas. The second phase included analyzing the suitability of each location for the development of solar, wind, and hydropower infrastructure. Then, we overlaid the location estimation results to obtain the optimal combination of locations for the construction of solar panels, wind farms, and hydropower plants in Southeast Asia. The third phase included processing the solar, wind, and hydropower potential models and comparing the power estimation results with the World Resources Institute (WRI) Global Power Plant Database^36^. Figure 10 shows the general structure of the work stages used in this study.

### Data utilized in this study

This study integrated geospatial data from various sources, such as tabular, vector, remote sensing, and climate modeling data, to analyze the potential of multi-renewable energy in Southeast Asia. A total of 20 datasets were used and grouped based on three types of data, namely, tabular and vector data, static raster data, and dynamic climate raster data. However, because the spatiotemporal resolutions of these data vary, a bilinear interpolation process was used to standardize the data resolution and ensure that the data used in the analysis all had a spatial resolution of 1 km. This research used tabular data and statistics on the energy demand per capita. Energy demand and supply data were taken from several sources, such as the Energy Information Administration, the World Bank, and Index Mundi-supported data from the Central Intelligence Agency (CIA)^40^. The data unit of energy consumption per capita was expressed in watt-hours, and the data was divided into rural and urban energy needs. Point vector data included airport point data, reservoir distribution data, and data on the distribution of existing power plants. Line vector data types included rivers, and polygon vectors include water bodies and protected areas. Geonode Global Airports Data^41^ is a global airport database with logistics information developed from various sources, such as OpenStreetMap and airports platform. The Geonode data were published in January 2019 and are regularly updated with input from the Logistics Cluster (LC) and the Logistics Capacity Assessment (LCA). The data for reservoirs and dams were obtained from the Global Reservoir and Dam Database (GranD v1.3)^42^. This product was developed from the Global Water System Project (GWSP), a collaborative effort to integrate global dam and reservoir data to support scientific studies. This product has 7320 datapoints in the form of buildings built up until 2016. Other data used in this study were obtained from the Global Power Plant Database^36^, which was developed by WRI experts and their partners. It includes comprehensive data related to power plants around the globe. The database covers approximately 35,000 power plants from 167 countries and includes thermal plants (e.g., coal, gas, oil, nuclear, biomass, waste, geothermal) and renewables (e.g., hydro, wind, and solar). Each datapoint provides information about plant capacity, generation, ownership, and fuel type. For rivers and water bodies, data from the United States Defense Mapping Agency's (DMA) operational navigation chart (ONC)^43^ was used. Buffering the data was necessary to reveal the distance from the water body to the study area. Additionally, the World Database on Protected Areas (WDPA) polygon^44^, a global database of protected terrestrial and marine areas, was used in this study. This product was developed as a joint project managed by the United Nation Environment Programme-World Conservation Monitoring Center (UNEP-WCMC).

The static raster data group included impervious surfaces, slope, stream order, flow accumulation, elevation, night light, land cover, population density, and stream flow. The Finer Resolution Observation and Monitoring of Global Land Cover (FROM-GLC)^45^ is the first product with a 30 m resolution urban land cover map. It was developed from Landsat image data with the “Exclusion/Inclusion” supervised classification algorithm in the Google Earth Engine platform. The FROM-GLC data includes information on changes in the global impervious surface area from 1985 to 2018. This product validation process uses a randomly selected sample unit with consistent accuracy of over 89%. Additionally, Advanced Land Observing Satellite-Digital Elevation Model (ALOS-DEM) data (Alos World 3D, 30 m; AW3D30) were used in this study to generate slopes with a 30 m resolution^46^. This data was developed by the Japan Aerospace Exploration Agency (JAXA) through the Panchromatic Remote-sensing Instrument for Stereo Mapping (PRISM). The data used to represent hydrological characteristics in this study were obtained from the Multi Error Removed Improved Terrain (MERIT) Hydro dataset^47^. This dataset is a product of flow direction at three arc-second resolutions (~ 90 m at the equator), developed from MERIT DEM data and water body datasets, including Global 1 arc-second Water Body Map (G1WBM), Global Surface Water Occurrence (GSWO), and OpenStreetMap, which includes data stream orders and flow accumulation. Comparisons of the MERIT product with similar products, such as those from the Global Runoff Data Center (GRDC), hydrological data and maps based on Shuttle Elevation Derivatives at Multiple Scales (HydroSHEDS), and Global River Widths from Landsat (GRWL), showed that the MERIT product does not have a significant error. In addition to ALOS and MERIT DEM data, this study also used elevation data from Shuttle Radar Topography Mission Global 1 arc second (SRTMGL1)^48^ with coverage over Asia and Australia. This product is void-filled using a combination of data from the Advanced Spaceborne Thermal Emission and Reflection Radiometer (ASTER), Global Digital Elevation Model 2 (GDEM2), United States Geological Survey (USGS) Global Multi-Resolution Terrain Elevation Data (GMTED), and USGS National Elevation Dataset (NED). The night light data used were from the Visible Infrared Imaging Radiometer Suite (VIIRS) Stray Light Corrected Nighttime Day/Night Band Composites (DNB)^49^. This data includes monthly average radiance composite images from 2014 to 2021 that were generated using nighttime data from VIIRS. The VIIRS DNB was developed by the Earth Observation Group (EOG) of the Colorado School of Mines, and it has a spatial resolution of 15 arc seconds. The land cover data used in this study were obtained from Copernicus Global Land Service-Land Cover 100 m (CGLS-LC100)^45^, which is global land cover map at a resolution of 100 m for the period 2015–2019. This data was developed through the CGLS Project for On-Board Autonomy–Vegetation (PROBA-V) 100 m time-series data, and it was validated using the Breaks for Additive Season and Trend (BFAST) algorithm at 28,321 sample sites. In addition to land cover data, this study considered supporting data including that on the population distribution. The population data product used in this study was the Wordpop Global Project Population Data (WGPPD)^50^. This data was developed based on a population census of administrative areas, which were aggregated into grid cells sized approximately 100 × 100 m through a machine learning approach (Random Forest-based dasymetric redistribution) based on population density with various geospatial covariate layers. Additional data considered in this study were obtained from the Global Streamflow Characteristics Dataset (GSCD)^51^. This data consisted of 17 streamflow characteristics, including the baseflow index, runoff coefficient, and flow timing with a spatial resolution of 15 km. The GSCD was developed through a data-driven (top-down) approach based on streamflow observations with an input source of about 7500 catchments around the globe.

Dynamic climate data have a monthly temporal resolution, which allows for an improved analysis of the contribution of monthly weather conditions to renewable energy conditions. The data included in this group were wind speed, air density, AOD, horizontal irradiance, cloud cover, precipitation, shortwave radiation, and temperature. The wind data used in this study were obtained from the Global Wind Atlas (GWA)^52^. This product is a collaboration between the Department of Wind Energy at the Technical University of Denmark (DTU Wind Energy) and the World Bank. The GWA has several data products, and the data selected from it for use in this study were wind speed and air density with a spatial resolution of 250 m. The air quality-related product was the daily Aerosol Optical Depth (AOD) MCD19A2 V6^53^. This AOD product was developed from a combination of MODIS Terra and Aqua data using a Multi-Angle Implementation of Atmospheric Correction (MAIAC) algorithm with a spatial resolution of 1 km. This product was developed using time-series analysis and a combination of pixel- and image-based processing. The Global Horizontal Irradiance (GHI) data from the Global Solar Atlas (GSA) database^54^ are the long-term daily averages of the total global horizontal irradiation, expressed in kWh/m^2^. The GHI data were collected until 2018 with a spatial resolution of 1 km. The GHI product was developed by Solargis and the World Bank. Its design was based on solar radiation data and temperature models, and it involves a validation process that is carried out by comparing field data from reference stations. The GHI product includes Cloud Optical Thickness (COT) data MYDAL2^55^, which measures how much sunlight can pass through clouds to reach the Earth's surface. These data were updated both daily and monthly, with a spatial resolution of 0.1°. To obtain precipitation data, the Climate Hazards Group InfraRed Precipitation with Station (CHIRPS) data product was used^56^. This product integrates satellite imagery with a spatial resolution of 0.05° with in situ station data to create a gridded rainfall time series. This dataset was developed using a 'smart' interpolation technique. An extended period of recorded precipitation was estimated based on infrared cold cloud duration observations. The validation process used the Global Precipitation Climatology Center (GPCC) product and independent station datasets. The final products are shortwave radiation and surface temperature, both of which use Climate Forecast System (CFSv2) data^57^, which was developed by the National Centers for Environmental Prediction (NCEP). This data is based on a climate model that represents the interactions between the Earth's atmosphere, oceans, land, and sea ice. CFSv2 has several product data with a spatial resolution of 20 km, including the shortwave radiation and surface temperature data used in this study.

### Energy demand estimation for urban and rural areas

In this study, the analysis of the estimated energy demand was associated with the distribution of the energy load used to determine the priorities for developing new renewable energy plants. This energy demand analysis was divided into two target areas to better estimate the results: urban and rural^58^. Statistical data on energy demand per capita in each country were integrated with statistical data on the country’s urban energy use and demand. The urban and rural area data in this study were obtained from a combination of impervious surface and night light data that separated urban and rural areas based on population density and level of economic activity. Furthermore, to estimate the amount of electricity consumed in urban and rural areas, we combined the statistical data on urban and rural energy consumption in each country with spatial data on population density. The final result obtained was the total electricity consumption for each data pixel in units of kilowatt-hours (kWh).

### Determining suitable areas for solar, wind, and hydropower development

Land suitability analyses for the development of renewable energy, specifically solar, wind, and hydropower, were carried out separately because each type of energy has different input data and parameters. The modeling of the area suitable for renewable energy was generally conducted using the multi-criteria analysis method^59^, which combines multiple input factors. All data were reclassified into five classes based on a literature review on Analytical Hierarchy Process (AHP) analysis. The AHP method was developed by Saaty et al.^60^, and it contributes to determining the weights used in multicriteria approaches. This method has the advantage of minimizing comparisons of complex decisions in pairs. Weighting is carried out using the pairwise comparison method, which is conducted through three major stages: compiling a pairwise comparison matrix, calculating the weights, and calculating the consistency ratio. After obtaining the weight of each parameter, the rated value for the parameter is multiplied by its importance to obtain a suitability index, as shown in Eqs. 1 and 2. In these equations, *W*_*i*_ is the weight of each criterion, *R*_*i*_ the class weight, and *S* is the regional suitability class.

1 $$ {\text{S }} = \frac{{\sum {\text{W}}_{{\text{i}}} {\text{R}}_{{{\text{ij}}}} }}{{\sum {\text{W}}_{{\text{i}}} }} $$

2 $$ S = (R_{1}^{\prime } W_{1} ) + (R_{2}^{\prime } W_{2} ) \, + \cdots + (R_{n}^{\prime } W_{n} ) $$

In the land suitability analysis for solar power plants, the parameters used included meteorological aspects of the GHI and COT data as well as geographical aspects, namely elevation, slope, and Land Use/Land Cover (LULC). To analyze the suitability of wind power plant locations, the parameters included geographical aspects, namely elevation, slope, LULC, and distance to rivers and water bodies. To analyze the suitability of hydro-energy power plant locations, the parameters used were stream order, LULC, and slope. After determining the suitable area for the three energy sources, constraint area exclusions were made, namely for protected areas for wind energy, airport locations, and dam locations for hydropower.

### Estimating energy potential for solar, wind, and hydropower

The next step was to calculate the estimated potential energy for each renewable energy source: solar, wind, and hydropower. To model the potential for solar energy, monthly average data on solar radiation (W/m^2^), temperature (K), AOD, and precipitation (mm/day) from January to December 2020 were used. There were three modeling steps. The energy potential produced by solar PV technology modeled the theoretical solar power potential, the effective solar power potential, and the solar PV-generated energy potential. The compelling power potential of solar panels was calculated using Eq. 3, which comes from the research of Principe and Takeuchi^12^ and is expressed in units of megawatts (MW). *A*_*cell*_ represents a solar panel installation pixel in a 1 km^2^ grid area. The efficiency of each solar cell was defined as 15% based on the assumption that the solar cells are made of crystalline silicon. In the equation, *R* represents the maximum solar radiation value for each month. In this study, the solar PV efficiency reduction analysis used temperature, rain, and AOD data. Equation 4 shows the algorithm for reducing the solar PV efficiency, with monthly average temperature calculated using a typical Nominal Operating Cell Temperature (NOCT) with a NOCT_max_ of 45 °C and a temperature efficiency coefficient of 0.094^61^. The value of *n* is the number of days per month. The reduction in solar PV efficiency due to AOD and rain was calculated using Eq. 5, where 0.035 is a constant representing the decrease in solar panel efficiency due to AOD and rain^62^. The value of *x*_*a,i*_ is the AOD content in the atmosphere in 1 month, while *x*_*r,i*_ is the number of rainy days in 1 month. *x*_*r,i*_ is calculated by a binary weighting of the rain data every day for 1 month, with a value of 1 if precipitation is > 7 mm/day and 0 if precipitation is < 7 mm/day.

3 $$ {\text{P}}_{{{\text{PV}}}}^{\prime } = {\text{A}}_{{{\text{cell}}}} \times \eta \times {\text{R}}^{\prime } \left( {1 - \underline{{\Delta \eta_{{\text{t}}} }} - \underline{{\Delta \eta_{{\text{d}}} }} } \right) $$

4 $$ \underline{{\Delta {\upeta }_{{\text{t}}} }} = 0.094\frac{{\mathop \sum \nolimits_{{{\text{i}} = 1}}^{{\text{n}}} {\text{LST}} - {\text{NOCT max}}}}{{\text{n}}} $$

5 $$ \underline{{\Delta {\upeta }_{{\text{d}}} }} = 0.035\frac{{\mathop \sum \nolimits_{{{\text{i}} = 1}}^{{\text{n}}} {\text{x}}_{{{\text{a}},{\text{i }}}} {\text{x}}_{{{\text{r}},{\text{i}}}} }}{{\text{n}}} $$

The estimation of potential wind energy was carried out in three stages: the development of a wind power density model, a technical analysis of the suitability model, and the development of a socio-economic model. In this study, the turbine energy potential was explicitly calculated for large-scale turbines with a height of 100 m. Data from the GWA were reclassified into class ranges to define the theoretical wind speed at an altitude of 100 m. The power calculation was based on the unit area or wind power density shown in Eq. 6, which was developed by Brown and Warne^63^. In this equation, *E*_*a*_ is the adequate power produced by the windmill (watts), *C*_*p*_ is the power coefficient in the form of air density (kg/m^3^), *D* is the diameter of the windmill (m), and *V* is the wind speed (m/s). For electricity generated by substitution into a wind energy conversion system^64^, Eq. 7 was used, where $C_{p}$ is a power coefficient of 0.4, $\eta_{tr}$ is a transmission efficiency of 0.95, $\eta_{g}$ is a generator efficiency of 0.85, and $\eta_{b}$ is a battery efficiency of 0.75. However, due to data limitation, the wind direction was not analyzed in this research.

6 $$ {\text{E}}_{{\text{a}}} = \frac{1}{2}{\text{C}}_{{\text{p}}} {\rho D}^{2} {\text{V}}^{3} $$

7 $$ \frac{{{\text{P}}_{{{\text{syst}}}} }}{{\text{A}}} = {\text{ C}}_{{\text{p}}} \times {\upeta }_{{{\text{tr}}}} \times {\upeta }_{{\text{b}}} \times \frac{1}{2}{\uprho } \times {\text{v}}^{3} $$

The energy potential of hydropower was calculated using Eq. 8, which was adapted from the research of Tarife et al.^27^ In this equation, *P* is the power produced (watts), *η* is the efficiency value of the turbine and generator, ${\rho  }$ is the density of water (1 kg/L), *g* is the value of gravity, *H* is the head (m), and *Q* is the river discharge (m^3^/s). The two main parameters (head and river discharge) were derived from Eq. 9, where $\alpha$ is the slope (degree) and $L$ is the average length of the diverted path (m). The head is the height difference between the potential point of a hydropower plant and a surrounding point within a certain radius or segment. This study obtained the head parameter by combining the ALOS DSM elevation model and the MERIT river network.

8 $$ P = \eta \rho {\text{gHQ}} $$

9 $$ {\text{H}} = {\text{tan }}\left( {\upalpha } \right) \times {\text{ L}} $$

### Comparison of the study results with the power plant energy database

The results of the suitability level analysis and potential energy estimation were compared with data from the WRI Global Power Plant Database^65^ to identify the accuracy of the calculations. The WRI database includes the location of power plants up to 2018 and information on the power generated in megawatts. Standardizing the data was necessary to accurately compare data from the models with data from the field. For example, because this study used satellite data represented in raster form with a resolution of 1 km^2^, it was essential to equalize the cross-sectional area of the power plants between the product and the field data. To determine the area of solar panels in the field, we used Google Earth to digitize the area of the solar panels after obtaining their location coordinates from the database. In the wind energy generation analysis, the resulting product was in the form of generated power; therefore, the results needed to be first transformed into energy by integrating the angular cross-sectional area and the adequate working time of the power plant. This process assumes that the power plant can operate for 24 h and is not affected by weather or day and night conditions. It was necessary to equalize the power unit, which refers to the field data with units of MW, and calculate the average power at points in the buffer area.

## Data Availability

Multi renewable energy suitability level and energy potential data products can be accessed at figshare repository in GeoTiff format: 10.6084/m9.figshare.21828273.v1.
